# Hard and Transparent Films Formed by Nanocellulose–TiO_2_ Nanoparticle Hybrids

**DOI:** 10.1371/journal.pone.0045828

**Published:** 2012-10-01

**Authors:** Christina Schütz, Jordi Sort, Zoltán Bacsik, Vitaliy Oliynyk, Eva Pellicer, Andreas Fall, Lars Wågberg, Lars Berglund, Lennart Bergström, German Salazar-Alvarez

**Affiliations:** 1 Materials and Environmental Chemistry, Stockholm University, Stockholm, Sweden; 2 Wallenberg Wood Science Center, KTH, Stockholm, Sweden; 3 Institució Catalana de Recerca i Estudis Avançats (ICREA) and Departament de Fsica, Universitat Autònoma de Barcelona, Bellaterra, Spain; 4 Departament de Fsica, Universitat Autònoma de Barcelona, Bellaterra, Spain; 5 Fibre and Polymer Technology, Royal Institute of Technology, KTH, Stockholm, Sweden; Argonne National Laboratory, United States of America

## Abstract

The formation of hybrids of nanofibrillated cellulose and titania nanoparticles in aqueous media has been studied. Their transparency and mechanical behavior have been assessed by spectrophotometry and nanoindentation. The results show that limiting the titania nanoparticle concentration below 16 *vol*% yields homogeneous hybrids with a very high Young’s modulus and hardness, of up to 44 GPa and 3.4 GPa, respectively, and an optical transmittance above 80%. Electron microscopy shows that higher nanoparticle contents result in agglomeration and an inhomogeneous hybrid nanostructure with a concomitant reduction of hardness and optical transmittance. Infrared spectroscopy suggests that the nanostructure of the hybrids is controlled by electrostatic adsorption of the titania nanoparticles on the negatively charged nanocellulose surfaces.

## Introduction

Organic–inorganic nanocomposites or hybrids have attracted much interest due to their current and potential applications as they can combine useful chemical, optical and mechanical characteristics. [1], [2] Traditionally, organic–inorganic nanocomposites have had a focus on the polymeric matrix, being e.g., formed from vinyl polymers, condensation polymers or polyolefins filled with relatively passive inorganic components such as layered silicates, i.e., montmorillonite or hectorite. [1], [2] With the strong movement towards biodegradable, renewable, sustainable, and carbon-neutral polymeric materials, it is also of importance to develop viable and facile production routes for nanocomposites using such biopolymers. In this respect, nanocellulose [3] is emerging as a cheap and sustainable polymeric material with useful functional properties such as tailored hydro/oleophilicity, optical transparency and remarkable mechanical performance both as films and aerogels. [4]–[9] The exploration of nanocellulose-nanoparticle hybrids is still relatively sparse but has increased pronouncedly since the pioneering report on multifunctional magnetic nanocellulose hybrids. [10] Recent studies have suggested various applications for different nanocellulose-inorganic hybrids: nanocrystalline cellulose-amorphous calcium carbonate hybrid films resemble biogenic materials such as dentin, [11] nanocellulose-clay nanopaper has shown good fire retardancy and gas barrier functions, [12] nanocellulose aerogels coated with titania using a CVD approach display a photoswitchable hydrophobicity [13] and oil adsorption, [14] and nanocellulose-silver hybrids were evaluated as potential antibacterial agents. [15] Moreover, it should be noted that other biopolymers such as silk, [16], [17] chitin, [18], [19] or collagen [20] also can be utilized in the production of organic-inorganic hybrids.

Titania-based materials are very attractive due to their inherent high refractive index and UV absorbing properties. For instance, titania-polymer hybrids have been prepared with conductive polymers, [21]–[23] polyacrylonitrile electrospun fibers [24], polyacrylonitrile and carbon nanotubes [25], [26], block co-polymers, [27]–[29] polystyrene beads, [30], [31] polyamide, [32] acrylic acid or PMMA, [33]–[35] silicates or siloxanes, [36] polyimides, [37] epoxies, [38] and polycations. [39].

In this work, we demonstrate the facile fabrication of nanocellulose-titania nanoparticles hybrids with high inorganic content by the adsorption of TiO2 (anatase) nanoparticles on wood-derived nanofibrillated cellulose. The nanostructure of the hybrids was characterized mainly by electron microscopy and the optical transparency and mechanical performance of the hybrids were evaluated using spectrophotometry and nanoindentation tests, respectively. We show that the effective Young’s modulus, hardness and transparency of the hybrids are determined by their nanostructure, in particular, by the homogeneity of the inorganic and organic components. The optimum range of inorganic content, where the modulus and hardness of the hybrids exceed that of pure nanocellulose and the transparency is high, is identified and the mechanisms for the nanocellulose-titania interactions and agglomeration are discussed.

## Materials and Methods

### Materials

Commercial TiO_2_ (anatase) nanoparticles were dispersed in a 0.1 M HCl aqueous solution with a stock concentration 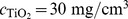. Nanofibrillated cellulose (NFC) was prepared by TEMPO oxidation of wood fibers according to a previously reported procedure which resulted in surface-functionalized fibrils with carboxylic groups with a total charge of 1.84 mmol/g. [6].

Aqueous dispersion of hybrids were prepared by adsorbing TiO_2_ nanoparticles onto NFC in an aqueous media. TiO_2_ nanoparticles, NFC (stock concentration 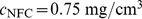) and water (Millipore, resistivity 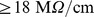) were mixed in different ratios, see Table 1, and their composition was also assessed using thermal analysis (see Supplementary Information, Figure S2). The dispersions were shaken for two hours and then the pH was adjusted to 8 with aqueous solutions of diluted NH3 and HCl.

**Table 1:** Composition of hybrids.

Sample	TiO2 ( 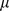 L)	NFC (mL)	H2O (mL)	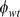 (*wt*% TiO2)	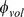 (*vol*% TiO2)
S1	0	1.25	5.6	0	0
S2	1.9	1.25	5.6	6	2
S3	3.8	1.25	5.6	11	4
S4	7.5	1.25	5.6	19	9
S5	15	1.25	5.6	32	16
S6	25	1.25	5.6	44	24
S7	35	1.25	5.6	53	30
S8	62.5	1.25	5.6	67	44

Films were prepared by depositing 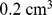 of an aqueous dispersion of the hybrids on circular glass slides (diameter 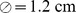) and placed in a Binder atmospheric chamber at 30°C and 50% relative humidity. The thickness of the obtained films was approximately 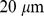. Alternatively, the aqueous dispersions of hybrids were centrifuged for 30 min at 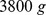 with a Hettich EBA 21 centrifuge, the supernatant was discarded and the remaining portion was freeze-dried at −40°C and a pressure of 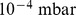 using a SRK GT2 freeze-drier.

### Morphological Characterization

#### Transmission Electron Microscopy (TEM)

Transmission electron microscopy (TEM) images of the titania nanocrystals were obtained using a JEOL JEM-2000 FX microscope equipped with a LaB6 filament operated at 200 kV (Cs = 3.4 mm, point resolution = 0.31 nm). The specimens were prepared by depositing a drop of a diluted dispersion of nanoparticles onto carbon-coated copper grids and allowing the solvent to evaporate. The images were recorded with a CCD camera (Keen View, SIS analysis, 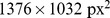, pixel size 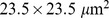). The particle length and width, 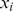 were manually measured on 200 nanoparticles from the TEM micrographs. The mean length or width, 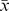, and its standard deviation, 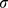, were determined by fitting the corresponding histogram (see supplementary Figure S1) with a Gaussian distribution function.

#### Scanning Electron Microscopy (SEM)

NFC and hybrid films were deposited on silicon chips (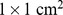) and mounted on aluminium stubs using carbon ink and coated with a thin carbon layer (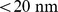). Alternatively, hybrid aerogels were glued on aluminum stubs using a double-sided carbon tape and also coated with a thin carbon layer (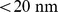). Scanning electron microscopy (SEM) images of the hybrids were acquired using a JEOL JSM-7401F field-emission gun microscope in secondary electron imaging mode at an accelerating voltage of 2 kV and probe current of 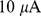 and working distance of 3 mm. The imaging of the films was carried out under the ‘GB High’ setting.

#### Atomic Force Microscopy (AFM)

Atomic force microscopy (AFM) images were recorded in tapping mode with the aid of a MultiMode instrument with Veeco NanoScope V controller using Veeco MPP-11100-10 silicon probes with nominal spring constants of 40 N/m. The force was kept minimal during scanning by routinely decreasing it until the tip left the surface and subsequently increasing it slightly to just regain contact. The scan rate was 0.5 to 2 lines per second. All images with 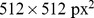 were analyzed with non-commercial software WSxM. [40].

### Spectroscopic Characterization

#### UV-Visible spectrophotometry

In-line transmittance spectra of the NFC and hybrids in the form of films on glass slides and as aqueous dispersions in the visible region (400–800 nm) were obtained with a Perkin-Elmer Lambda 19 UV-Vis-NIR spectrophotometer using a clean glass slide as a background. The aqueous dispersions were filled in a 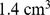 semi-micro rectangular quartz cuvette using Millipore water as background.

#### Infrared spectroscopy

Infrared (IR) spectra of NFC, TiO_2_ and NFC-TiO_2_ hybrids materials were measured on a Varian 670-IR FTIR spectrometer, equipped with an attenuated total reflection (ATR) detection device (Goldengate by Specac) with a single reflection diamond ATR element. 32 scans were accumulated in the spectral region of 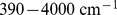 with a spectral resolution of 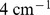. To maximize the signal of the carboxylic band the pH of aqueous dispersions of NFC and was adjusted to pH = 3 with few microliters of diluted HCl and NH3 prior to contacting them with the aqueous dispersions of TiO_2_. The pH of the pristine aqueous dispersions of TiO_2_ was also adjusted to pH = 3 before the measurements. The materials were then freeze-dried before the measurements using the protocol previously described.

### Mechanical Characterization

The mechanical properties of the hybrids were evaluated using a Fischer-Cripps Laboratories Ultra-Micro-Indenter system (UMIS) equipped with a Berkovich pyramidal-shaped diamond tip. The value of maximum applied force was chosen to be 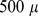
N to ensure that the maximum penetration depth was kept well below one tenth of the overall film thickness (a necessary condition to avoid having an influence of the substrate on the measured mechanical properties of the film). [41] The thermal drift during nanoindentation was kept below 0.05 nm/s. Proper corrections for the contact area (calibrated with a fused quartz specimen), instrument compliance, and initial penetration depth were applied. The hardness, 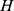, and effective reduced Young’s modulus, 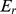, values were derived from the load-displacement curves at the beginning of the unloading segment using the method of Oliver and Pharr. [42] From the initial unloading slope, the contact stiffness, 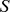, was determined as 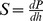 where 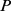 and 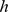 denote, respectively, the applied load and the penetration depth during nanoindentation. The effective reduced Young’s modulus was evaluated based on its relationship with the contact area, *A*, and contact stiffness 
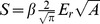
. Here, 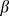 is a constant that depends on the geometry of the indenter (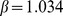 for a Berkovich indenter), and 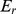 is defined as 
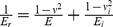
. [43] The reduced modulus takes into account the elastic displacements that occur in both the specimen, with effective Young’s modulus *E* and Poisson’s ratio 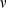, and the diamond indenter, with elastic constants 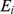 and 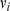. Note that for diamond, 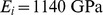 and 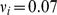. Remarkably, for most materials, including NFC or TiO_2_, where 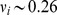 [44] and 0.27, [45], [46] respectively, the contribution of the tip to 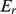 is almost negligible, i.e., 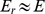 (5% overestimation). The hardness was calculated as 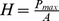 where 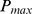 is the maximum load applied during nanoindentation. Finally, the elastic recovery was evaluated as the ratio between the elastic and the total (plastic + elastic) energies during nanoindentation, 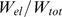. These energies were calculated from the nanoindentation experiments as the areas between the unloading curve and the *x*-axis (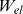) and between the loading curve and *x*-axis (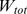). [43] The results presented here represent the statistical average of a set of 50 indentations for each sample, whereas up to 200 indentations were carried out on the samples with low inorganic content (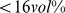).

## Results and Discussion

**Figure 1:**
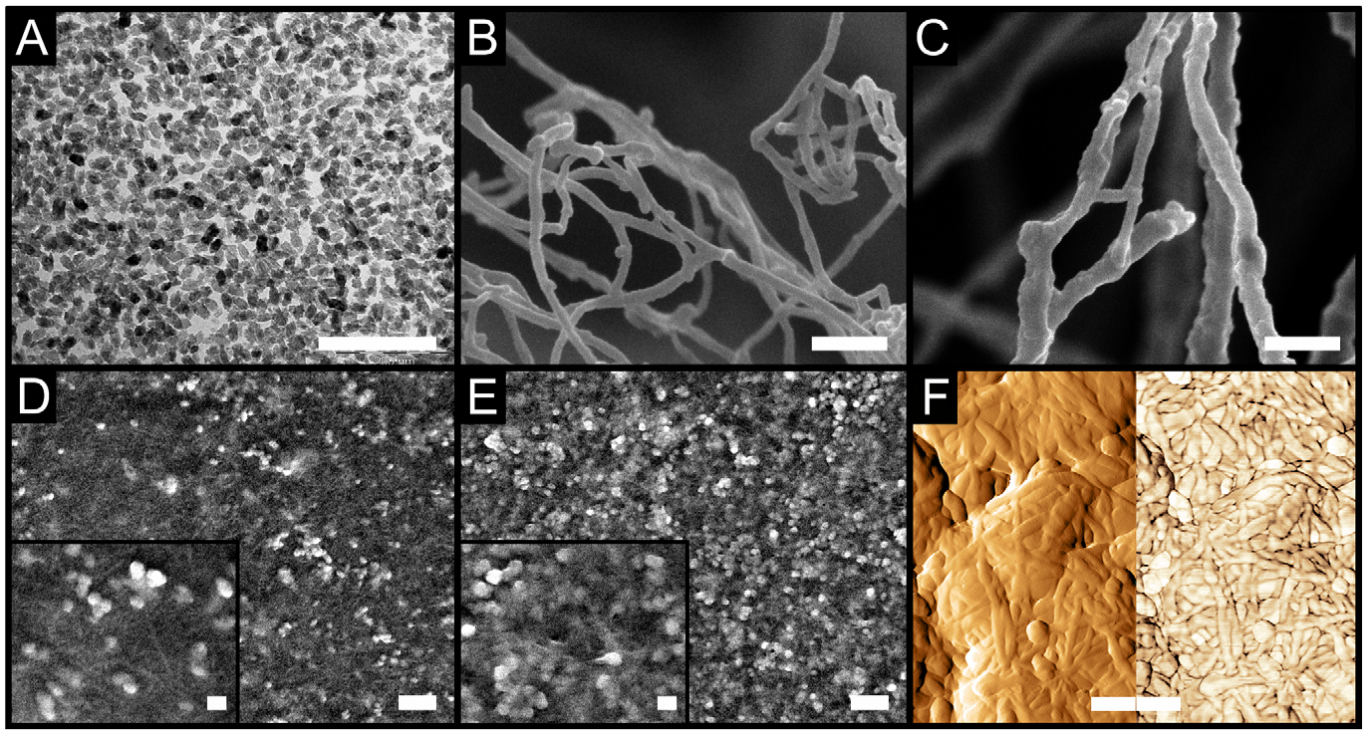
Morphological characterization of the materials. (A) TEM micrograph of titania nanoparticles. (B-E) SEM micrographs of freeze-dried samples corresponding to (B) pristine nanofibrillated cellulose, NFC and (C) a hybrid composed of NFC-TiO_2_ with a 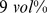 of inorganic content (S4); films of NFC-TiO2 hybrids deposited on silicon wafers with (D) 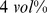 (S3) and (E) 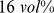 of inorganic content (S5). (F) Derivative and phase AFM images of the hybrids shown in (E). The scale bars represent 200 nm (50 nm in the insets).

Figures 1a and 1b show electron microscopy images of the hybrid constituents, i.e., TiO_2_ nanoparticles and NFC fibers, respectively. Analysis of the TEM images showed that the TiO_2_ particles had a length 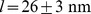 and a width 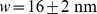, i.e., an aspect ratio 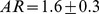 (see Supplementary Information, Figure S1). The cellulose nanofibrils had a width distribution between 3–5 nm and length of 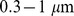. However, during the adsorption of TiO_2_ the fibrils tend to agglomerate somewhat and form bundles with thickness between 10–20 nm, as can be see in Figure 1c which shows a SEM micrographs of the hybrid aerogel with 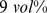 of inorganic content. Hybrids of NFC and TiO_2_ nanoparticles were prepared also as films. Representative SEM images of hybrid films with low (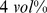 TiO_2_) and medium (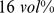 TiO_2_) inorganic content are shown in Figures 1d and 1e, respectively. The images show that the amount of fibers on the surface decreases with an increase of the fraction of nanoparticles. Films with a large amount of TiO_2_ nanoparticles (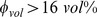) display an even granular surface. AFM images (Figure 1f) show that the inorganic nanoparticles are distributed on the surface of the films and also in between the fibrils.

**Figure 2:**
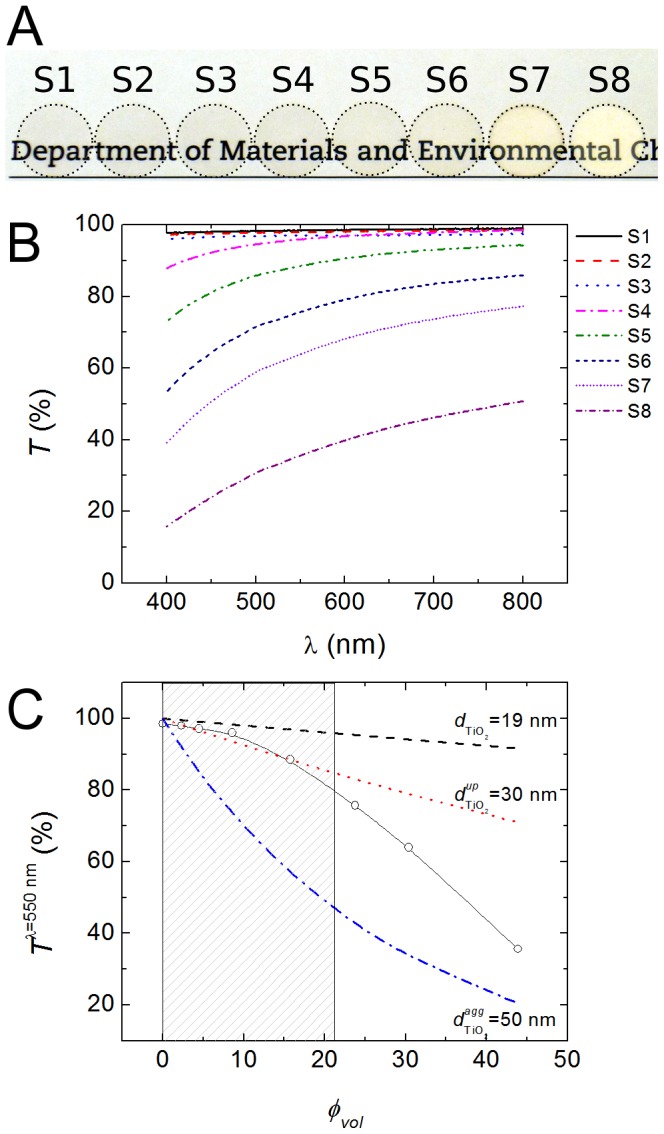
Optical characterization of the hybrids. (A) Optical photograph of NFC-TiO_2_ hybrid films deposited on glass slides (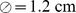, indicated by dashed contours). (B) Transmittance, 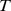, of the NFC and hybrid films with an increasing TiO_2_ content, 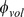 (see Table 1), as a function of the wavelength, 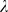. (C) Transmittance of the NFC and hybrid films at 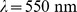 as a function of the TiO_2_ content, 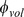. The symbols represent the experimental data whereas the three lines correspond to the calculated transmittance of films composed of particles with three different sizes, i.e., 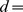 19, 30, and 50 nm according to Eq. (1), see text for details. The hatched area shows the region where the hybrids display high transparency (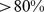).

Figure 2a shows a photograph of the films deposited on glass. The films are transparent at low inorganic content but tend to become milky as the concentration of nanoparticles increases, suggesting that light scattering becomes increasingly important. The optical transmittance, 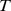, in the visible region of the different film samples with different inorganic content is shown in Figure 2b. The figure shows that the NFC film has a high optical transmittance over the visible range, as expected from its low absorption coefficient [47] and smoothness of the films. The hybrids with relatively low concentration of inorganic nanoparticles have a high transmittance in the visible area which decreases toward the ultraviolet region, when the bandgap of anatase is approached. In the case of hybrids, in the absence of significant absorption, the transmitted light across a hybrid film can be described using the Rayleigh formalism for scattering, as indicated by Eq. (1). [48]

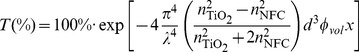
(1)where 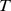 is the transmittance, 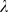 the wavelength, 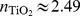 and 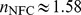 the average refractive indices of anatase [49] and cellulose [47], respectively, 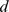 the diameter of the particles, and 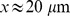 the thickness of the film. Note that the model assumes that the NFC matrix is dense and nonporous and that the particles are point scatterers much smaller than the wavelength, i.e., 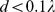.

The equation shows that the transmittance decreases with an increase of: the concentration of nanoparticles, the particle size, the difference of refractive indices, and the thickness of the films, and with a decrease of the wavelength. Indeed, as anatase and cellulose have a very low absorption in the visible region it is possible to use Eq. (1) to model the response of the hybrids using the experimental data. Figure 2c shows the optical transmittance of the films at 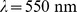. Plotted along the experimental points are the calculated transmittance for three particle sizes: (i) particle diameter with an equivalent particle volume as the TiO_2_ nanoparticles used for the fabrication of the hybrids (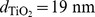); (ii) particles with a diameter equal to the upper limit of the experimental particle size (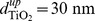); and (iii) particles with a diameter similar to the observed agglomerates 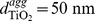. The experimentally observed transmittance of the hybrid films with an inorganic content 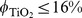 can be well described within the boundaries described by (i) and (ii) (hatched region). Alternatively, the transmittance of the hybrids with 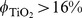 is between the boundaries defined by (ii) and (iii), suggesting that the number of agglomerates becomes increasingly important. Notice that both the reflectivity [39] and the surface roughness of the films [50] also contribute to a slightly lowered transmittance.

Previous work on the fabrication of poly(vinyl alcohol)-TiO_2_ (rutile) nanoparticle (PVAL-TiO_2_) nanocomposites have also demonstrated high transparency in hybrid films. [51] The hybrids, with a thickness 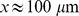, were formed in a similar fashion as the ones described in the present work, i.e., by the ex-situ nucleation of nanoparticles (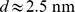) and their subsequent mixing and drying with the polymer. As a comparison, PVAL-TiO2 hybrids with a 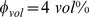 showed a 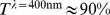. Sasaki et al. prepared poly(diallyldimethylammonium chloride)–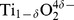 (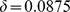) nanoplatelet nanocomposite films using the layer-by-layer technique (LbLTiO2). [39] The hybrids were composed of alternating layers of polymer and 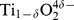 nanoplatelets (thickness ca. 1.2 nm, lateral dimensions in the sub-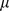
m regime). However, the LbLTiO2 hybrids showed a substantial reflectivity which decreased the optical transmittance. For instance, a 10-repeat multilayer with a thickness of 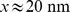 showed a reduced 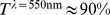. Regarding the current work, it is interesting to note that despite the relatively large size of the anatase nanoparticles and the fibrillated structure of the nanocellulose, the transparency of the hybrids is very high and comparable to those systems prepared from smaller particles.

**Figure 3:**
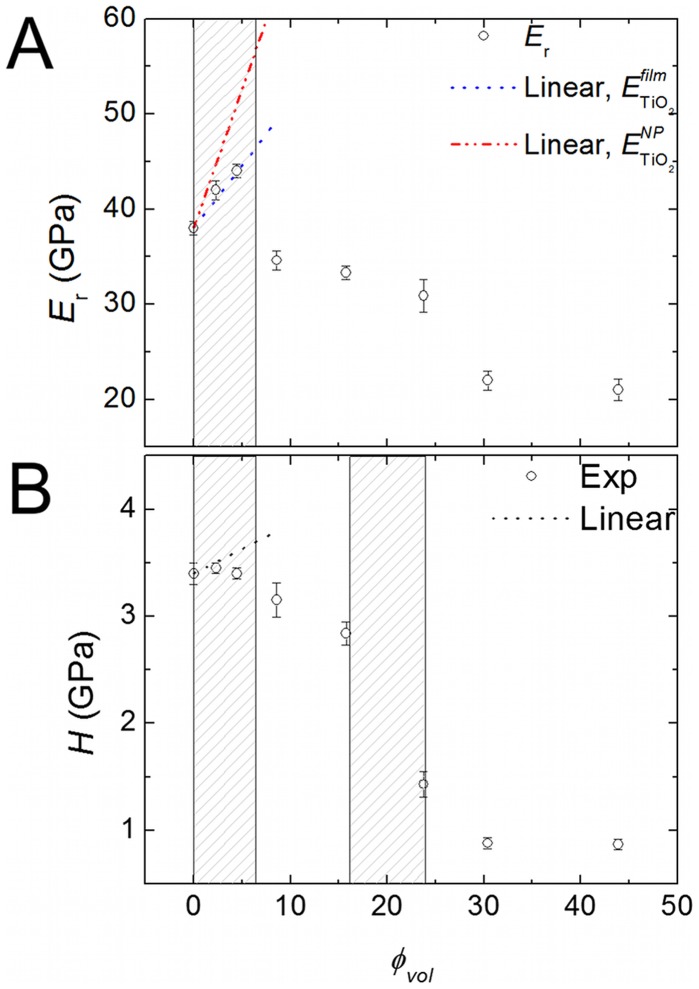
Variation of the mechanical properties with the composition of the hybrids. (A) Effective reduced Young’s modulus, 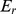, of hybrids with increasing TiO_2_ content, 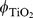. The symbols correspond to the experimental values and the error bars to the standard deviation from several indentations (see text). The dotted and dash-dotted lines correspond to a linear variation of 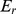 from the rule of mixtures (see text) using 
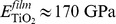
 [53], [54] or 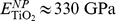, [45] respectively; and 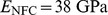 (this work). The hatched region indicates the region where the linear increase is observed. (B) Variations of hardness, 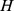, as a function of 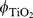. The dotted line in (b) corresponds to a linear variation of 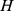 (see text) assuming 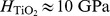 [53], [54] and 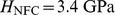 (this work). The hatched regions represent the areas of high hardness and the transition region toward low hardness.

The mechanical behavior of the films was tested using nanoindentation measurements where the typical load-displacement nanoindentation curves and AFM images of the indents are shown in the supplementary information (Figures S4 and S5). Figure 3 shows the effective reduced Young’s modulus, 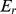, and hardness, 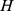, of the films as a function of inorganic content, 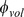. The 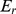 value corresponding to NFC is in close agreement with the reported values of the transversal Young’s modulus of native cellulose. [52] The addition of a small amount of TiO_2_ nanoparticles resulted in a slight increase of 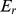. This initial increase of 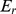 as 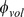 increases can be described with a simple linear rule of mixtures, i.e., 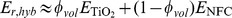. Figure 3a includes the estimates for hybrids with low 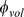 (hatched area) using the experimentally reported values for the elastic modulus of anatase thin films (nanoindentation), 
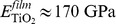
, [53], [54] and nanoparticles (high pressure X-ray), 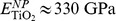. [45] The experimental value obtained for sample S1 is assigned to pure NFC, i.e., 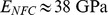. However, as the concentration of nanoparticles increased further the 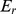 value of the hybrid films decayed abruptly. Indeed, at higher concentration of nanoparticles, 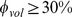, the films became looser and compliant, leading to a decrease of 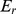. This behavior suggests that the bonding and microstructure of the hybrids change significantly with increasing anatase content. Remarkably, the NFC and hybrid films with TiO_2_ concentrations up to 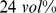 had extraordinarily high effective Young’s modulus when compared to organic-inorganic hybrids previously reported (see [41] and references therein) and some high-performance lightweight materials, such as magnesium [55] or concrete. [56], [57] The linear increase in 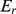 at low TiO_2_ additions strongly indicates that the inorganic nanoparticles are homogeneously distributed and bonded to the NFC network, thus increasing the modulus (and hardness) with increasing amount of the stiff and hard constituent. At a critical concentration, the homogeneity of the hybrid and the anatase nanoparticle distribution decreases. Recent reports have indeed shown that non-sintered films composed of anatase TiO_2_ nanoparticles have Young’s moduli as low as 22.5 GPa. [58], [59] This strong dependence of the mechanical behavior on the TiO_2_ nanoparticle content is much more evident in Figure 3b where the hardness, 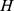, of the NFC and hybrid films is depicted. The hardness of the NFC and hybrids with 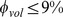 is roughly constant about 3.4 GPa (first hatched area). As the concentration of nanoparticles further increases there is a sharp decrease of the hardness values at volume fractions 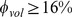 (second hatched area), with 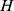 falling below 1 GPa. Note that AFM analysis of the indentations shows little pile-up and sink-in at high TiO_2_ contents (30 *vol%*) thus having a reduced influence on the overall trends shown in Figure 3 see supplementary information Figure S5).

**Figure 4:**
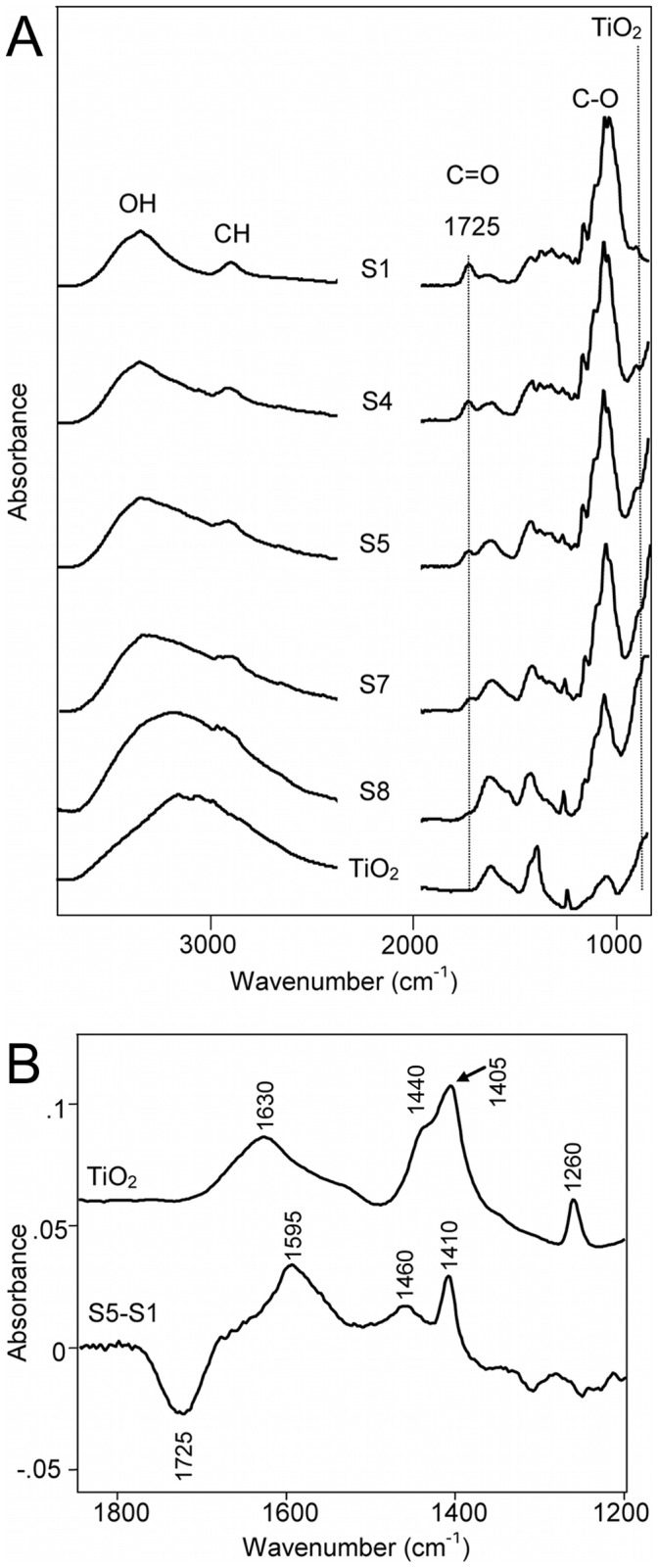
Infrared spectra of the different samples as a function of TiO_2_ content. (A) The position of some of the bands are indicated in the figure. The band at 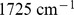 corresponds to the the C = O stretching of the carboxylic group on the surface of the cellulose. [62]–[64] The bands at 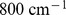 correspond to the anatase. Note that the different bands in the spectra were normalized using the intense C-O band region of the cellulose at around 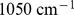. (B) Infrared spectra of a) TiO_2_ nanoparticles and b) difference spectrum of the composite material with 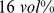 TiO_2_ and pure cellulose using the C-O band of the cellulose at 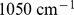 as internal reference. The band at 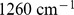 is used to normalize the TiO_2_ spectrum, to clarify the differences (see text).

We have used IR spectroscopy to obtain more information on the bonding and interaction between the inorganic nanoparticles and the nanocellulose. The IR spectra are shown in Figure 4a for NFC, TiO_2_ nanoparticles, and the hybrids with different inorganic content. The broad band at the low frequency end of the spectra (also partially related to the librational mode of adsorbed water) is assigned to the Ti-O band and increases with the TiO_2_ content. To facilitate the analysis of the interactions between the nanoparticles and the NFC, the nanocellulose and the hydroxyl groups on the surface of the titania nanoparticles were protonated prior to the formation of the hybrid. Hence, by observing the C = O stretching region of the (protonated) carboxylic groups on the surface of the fibrils it is possible to correlate the reaction between the fibrils and the positively charged TiO_2_ nanoparticles. Figure 4a shows that a decrease in the intensity of carboxyl band corresponding to the acidic C = O (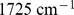) decreases with an increase of the concentration of TiO_2_. The formation of an ester between the carboxylic group of the nanocellulose and the hydroxyl groups on the surface of the nanoparticles was excluded as no C = O band was detected at higher frequencies than that corresponding to the acidic C = O. Using a difference spectra, the bands that take part in the NFC-TiO_2_ interactions are readily observed. Figure 4b shows the spectrum of freeze-dried TiO_2_ nanoparticles and the difference spectra of a hybrid with 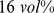 TiO_2_ from which the spectra of NFC was subtracted. The *negative* band at 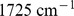 shows the decrease in the amount of carboxyl groups whereas the *positive* band at 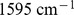 (antisymmetric stretching) suggests an increase in the amount of carboxylate group. Note that the band at 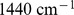 is due to ammonium ions, [60] whereas the bands at 1405 and 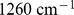 in the spectrum of TiO_2_ are likely to correspond to nitrate and nitrite ions [60] arising from the photooxidation of ammonia [61] used during pH adjustment as these bands do not appear in the IR spectrum of pristine TiO_2_ (see supplementary information Figure S3). The bands at 1460 and 1410 cm^−1^ could arise from the symmetric stretching of carboxylate ions, although the presence of artifacts during spectral subtraction cannot be ruled out.

Analysis of the spectra suggests that the nanocellulose and the nanoparticles interact through electrostatic interactions between the dissociated carboxylic group and the positively charged groups on the nanoparticles, i.e., 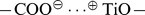. Other groups have prepared polymer-titania hybrids using a silane groups as grafting agent, resulting in a covalent modification of titania nanoparticles(e.g., a Ti–O–Si bond) formed in-situ, [23], [33], [35] whereas the the formation of polyaniline-titania probably also proceeds via electrostatic interactions. [21], [22] The formation of hybrids using ex-situ synthesized nanoparticles allow for a larger range of inorganic content that is accessible. However, the results also suggest that a careful balance of the electrostatic interactions between the nanocellulose and titania nanoparticles and their dispersability in aqueous media is key to ensure optimized optical and mechanical properties.

### Conclusions

Hybrids composed of nanofibrillated cellulose and anatase nanoparticles with variable inorganic content were fabricated through the adsorption of ex-situ prepared nanoparticles. Electron microscopy shows that the homogeneity of the hybrids decreases towards high concentration of nanoparticles. The reduction in homogeneity resulted in a reduced hardness and reduced optical transparency. Infrared spectroscopy demonstrated that the nanocellulose and nanoparticles are bound through electrostatic interactions and not through the formation of covalent bonds. The hybrids with an optimized inorganic content presented in the current work showed extraordinary optical and mechanical properties, with high transmittances in the visible region and high effective Young’s modulus and hardness superior to previously reported materials. These properties suggest a potential use of nanocellulose-based hybrids as transparent coatings where high wear resistance and UV activity are required.

## Supporting Information

Figure S1 **Titanium dioxide nanoparticles and their size distribution.** (left) TEM image of titanium dioxide nanoparticles and (right) histograms built from the manual determination of particle length and width. The lines correspond to a fit with a Gaussian distribution function.
(TIF)

Figure S2 **Thermogravimetric analysis of the samples.** TGA was performed on a Perkin Elmer Thermogravimetric Analyzer TGA7. Ca. 1 mg of the different hybrids (freeze-dried from the solutions) was filled in a platinum cup and analyzed under technical air from 30–900°C at a heating rate of 5 K/min. (left) The initial weight loss up to 200°C corresponds to the release of adsorbed water. The second weight loss from 200–500°C is due to the removal of NFC. (right) Derivative of the mass loss.
(TIF)

Figure S3
**Infrared spectra of TiO_2_ nanoparticles before (b) and after (a) addition of ammonium ions.** The band at 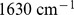 is due to the bending mode of adsorbed water, the one at 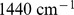 corresponds to the deformation vibration of ammonium ions, whereas the bands at 1405 and 1260 cm^−1^ are likely belong to the asymmetric stretching of nitrate and nitrite ions.(TIF)

Figure S4
**Load – displacement curves.** Indentation curves corresponding to hybrids with different compositions deposited as films on glass substrates. The thickness of the films is ca. 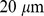.(TIF)

Figure S5 **Atomic force microscopy derivative images.** Indentations performed on samples S2 (left) and S7 (right). The circles highlight the indentations, where no pronounced pile-up is observed.
(TIF)

Table S1 **Additional mechanical properties of the hybrid NFC/TiO_2_ films obtained from nanoindentation experiments.** Parentheses indicate the standard deviation in the last digits.
(PDF)

## References

[1] Alexandre M, Dubois P (2000) Polymer-layered silicate nanocomposites: preparation, properties and uses of a new class of materials. Mater Sci Eng R 28: 1–63.

[2] Ray SS, Okamoto M (2003) Polymer/layered silicate nanocomposites: a review from preparation to processing. Prog Polym Sci 28: 1539–1641.

[3] Klemm D, Kramer F, Moritz S, Lindström T, Ankerfors M, et al (2011) Nanocelluloses: A new family of nature-based materials. Angew Chem Int Ed 50: 5438–5466. 10.1002/anie.201001273 21598362

[4] Gross RA, Kalra B (2002) Biodegradable polymers for the environment. Science 297: 803–807. 12161646 10.1126/science.297.5582.803

[5] Beecher JF (2007) Organic materials: Wood, trees and nanotechnology. Nat Nanotechnol 2: 466–467. 18654341 10.1038/nnano.2007.239

[6] Isogai A, Saito T, Fukuzumi H (2011) Tempo-oxidized cellulose nanofibers. Nanoscale 3: 71–85. 20957280 10.1039/c0nr00583e

[7] Sehaqui H, Salajkova M, Zhou Q, Berglund LA (2010) Mechanical performance tailoring of tough ultra-high porosity foams prepared from cellulose i nanofiber suspensions. Soft Matter 6: 1824–1832.

[8] Sehaqui H, Zhou Q, Ikkala O, Berglund LA (2011) Strong and tough cellulose nanopaper with high specific surface area and porosity. Biomacromolecules 12: 3638–3644. 21888417 10.1021/bm2008907

[9] Cervin NT, Aulin C, Wågberg L, Larsson T (2011) Hydrophobic aerogels from nanofibrillated cellulose (nfc) with tunable oleophilicity. In: Abstracts of Papers of the American Chemical Society. p.241.

[10] Olsson R, Samir MA, Salazar-Alvarez G, Ström V, Belova L, et al (2010) Making exible magnetic aerogels and stiff magnetic nanopaper using cellulose nanofibrils as templates. Nat Nanotechnol 5: 584–588. 20676090 10.1038/nnano.2010.155

[11] Gebauer D, Oliynyk V, Salajkova M, Sort J, Zhou Q, et al (2011) A transparent hybrid of nanocrystalline cellulose and amorphous calcium carbonate nanoparticles. Nanoscale 3: 3563–3566. 21850350 10.1039/c1nr10681c

[12] Liu A, Walther A, Ikkala O, Belova L, Berglund LA (2011) Clay nanopaper with tough cellulose nanofiber matrix for _re retardancy and gas barrier functions. Biomacromolecules 12: 633–641. 21291221 10.1021/bm101296z

[13] Kettunen M, Silvennoinen RJ, Houbenov N, Nykänen A, Ruokolainen J, et al (2011) Photoswitchable superabsorbency based on nanocellulose aerogels. Adv Funct Mater 21: 510–517.

[14] Korhonen JT, Kettunen M, Ras RHA, Ikkala O (2011) Hydrophobic nanocellulose aerogels as oating, sustainable, reusable, and recyclable oil absorbents. ACS Appl Mater Interfaces 3: 1813–1816. 21627309 10.1021/am200475b

[15] Díez I, Eronen P, Österberg M, Linder MB, Ikkala O, et al (2011) Functionalization of nanofibrillated cellulose with silver nanoclusters: Fluorescence and antibacterial activity. Macromol Biosci 11: 1185–1191. 21728237 10.1002/mabi.201100099

[16] Wong Po Foo C, Patwardhan SV, Belton DJ, Kitchel B, Anastasiades D, et al (2006) Novel nanocomposites from spider silk-silica fusion (chimeric) proteins. Proc Nat Acad Sci USA 103: 9428–9433. 16769898 10.1073/pnas.0601096103 PMC1476692

[17] Kharlampieva E, Kozlovskaya V, Gunawidjaja R, Shevchenko VV, Vaia R, et al (2010) Flexible silk-inorganic nanocomposites: From transparent to highly reective. Adv Funct Mater 20: 840–846.

[18] Alonso B, Belamie E (2010) Chitins-ilica nanocomposites by self-assembly. Angew Chem Int Ed 49: 8201–8204. 10.1002/anie.201002104 20865706

[19] Ifuku S, Morooka S, Morimoto M, Saimoto H (2010) Acetylation of chitin nanofibers and their transparent nanocomposite films. Biomacromolecules 11: 1326–1330. 20359173 10.1021/bm100109a

[20] Thanikaivelan P, Narayanan NT, Pradhan BK, Ajayan PM (2012) Collagen based magnetic nanocomposites for oil removal applications. Sci Rep 2: 230. 22355744 10.1038/srep00230 PMC3262048

[21] Feng W, Sun E, Fujii A, Wu H, Niihara K, et al (2000) Synthesis and characterization of photoconducting polyaniline-TiO2 nanocomposites. Bull Chem Soc Jpn 73: 2627–2633.

[22] Li X, Chen W, Bian C, He J, Xu N, et al (2003) Surface modification of TiO_2_ nanoparticles by polyaniline. Appl Surf Sci 217: 16–22.

[23] Li J, Zhu L, Wu Y, Harima Y, Zhang A, et al (2006) Hybrid composites of conductive polyaniline and nanocrystalline titanium oxide prepared via self-assembling and graft polymerization. Polymer 47: 7361–7367.

[24] Drew C, Liu X, Ziegler D, Wang X, Bruno FF, et al (2003) Metal oxide-coated polymer nanofibers. Nano Lett 3: 143–147.

[25] Kedem S, Schmidt J, Paz Y, Cohen Y (2005) Composite polymer nanofibers with carbon nanotubes and titanium dioxide particles. Langmuir 21: 5600–5604. 15924496 10.1021/la0502443

[26] Banerjee S, Wong SS (2002) Synthesis and characterization of carbon nanotube-nanocrystal heterostructures. Nano Lett 2: 195–200.

[27] Sun Z, Kim D, Wolkenhauer M, Bumbu G, Knoll W, et al (2006) Synthesis and photoluminescence of titania nanoparticle arrays templated by block-copolymer thin films. Chem Phys Chem 7: 370–378. 16389600 10.1002/cphc.200500340

[28] Bartl MH, Boettcher SW, Frindell KL, Stucky GD (2005) 3-D molecular assembly of function in titania-based composite material systems. Acc Chem Res 38: 263–271. 15835873 10.1021/ar040177o

[29] Coakley KM, McGehee MD (2003) Photovoltaic cells made from conjugated polymers infiltrated into mesoporous titania. Appl Phys Lett 83: 3380–3382.

[30] Zhang M, Gao G, Li C, Liu F (2004) Titania-coated polystyrene hybrid microballs prepared with miniemulsion polymerization. Langmuir 20: 1420–1424. 15803728 10.1021/la030183d

[31] Caruso RA, Susha A, Caruso F (2001) Multilayered titania, silica, and laponite nanoparticle coatings on polystyrene colloidal templates and resulting inorganic hollow spheres. Chem Mater 13: 400–409.

[32] Kwak S, Kim SH, Kim SS (2001) Hybrid organic/inorganic reverse osmosis (RO) membrane for bactericidal anti-fouling. 1. preparation and characterization of TiO_2_ nanoparticle self-assembled aromatic polyamide thin-film-composite (TFC) membranes. Environ Sci Technol 35: 2388–2394. 11414050 10.1021/es0017099

[33] Chen WC, Lee SJ, Lee LH, Lin JL (1999) Synthesis and characterization of trialkoxysilane-capped poly(methyl methacrylate)-titania hybrid optical thin films. J Mater Chem 9: -.

[34] Davis SA, Breulmann M, Rhodes KH, Zhang B, Mann S (2001) Template-Directed assembly using nanoparticle building blocks: A nanotectonic approach to organized materials. Chem Mater 13: 3218–3226.

[35] Lee LH, Chen WC (2001) High-refractive-index thin films prepared from trialkoxysilane-capped poly(methyl methacrylate)titania materials. Chem Mater 13: 1137–1142.

[36] Schmidt H, Jonschker G, Goedicke S, Mennig M (2000) The sol-gel process as a basic technology for nanoparticle-dispersed inorganic-organic composites. J Sol-Gel Sci Technol 19: 39–51.

[37] Yoshida M, Lal M, Kumar N, Prasad P (1997) TiO2 nano-particle-dispersed polyimide composite optical waveguide materials through reverse micelles. J Mater Sci 32: 4047–4051.

[38] Ng C, Ash B, Schadler L, Siegel R (2001) A study of the mechanical and permeability properties of nano-and micron-TiO2, filled epoxy composites. Adv Comp Lett 10: 101–111.

[39] Sasaki T, Ebina Y, Tanaka T, Harada M, Watanabe M, et al (2001) Layer-by-layer assembly of titania nanosheet/polycation composite films. Chem Mater 13: 4661–4667.

[40] Horcas I, Fernández R, Gómez-Rodríguez JM, Colchero J, Gómez-Herrero J, et al (2007) Wsxm: A software for scanning probe microscopy and a tool for nanotechnology. Rev Sci Instrum 78: 013705. 17503926 10.1063/1.2432410

[41] Mammeri F, Bourhis EL, Rozes L, Sanchez C (2005) Mechanical properties of hybrid organicinorganic materials. J Mater Chem 15: 3787–3811.

[42] Oliver W, Pharr G (1992) An improved technique for determining hardness and elastic modulus using load and displacement sensing indentation experiments. J Mater Res 7: 1564–1583.

[43] Fischer-Cripps AC (2004) Nanoindentation. Springer.

[44] Zimmermann T, Pöhler E, Schwaller P (2005) Mechanical and morphological properties of cellulose fibril reinforced nanocomposites. Adv Eng Mater 7: 1156–1161.

[45] Chen B, Zhang H, Dunphy-Guzman KA, Spagnoli D, Kruger MB, et al (2009) Size-dependent elasticity of nanocrystalline titania. Phys Rev B 79: 125406.

[46] CERAM Research Ltd. Titanium dioxide. Available: http://www.azom.com/details.asp?ArticleID=1179. Accessed 2011 Oct 11.

[47] Nogi M, Handa K, Nakagaito AN, Yano H (2005) Optically transparent bionanofiber composites with low sensitivity to refractive index of the polymer matrix. Appl Phys Lett 87: 243110.

[48] Krug H, Schmidt H (1994) Organic-inorganic nanocomposites for micro optical applications. New J Chem 18: 1125–1134.

[49] Gonzalez RJ, Zallen R, Berger H (1997) Infrared reectivity and lattice fundamentals in anatase TiO2s. Phys Rev B 55: 7014–7017.

[50] Larena A, Millán F, Pérez G, Pinto G (2002) Effect of surface roughness on the optical properties of multilayer polymer films. Appl Surf Sci 187: 339–346.

[51] Nussbaumer RJ, Caseri WR, Smith P, Tervoort T (2003) Polymer-TiO_2_ nanocomposites: A route towards visually transparent broadband UV filters and high refractive index materials. Macromol Mater Eng 288: 44–49.

[52] Lahiji RR, Xu X, Reifenberger R, Raman A, Rudie A, et al (2010) Atomic force microscopy characterization of cellulose nanocrystals. Langmuir 26: 4480–4488. 20055370 10.1021/la903111j

[53] Zywitzki O, Modes T, Sahm H, Frach P, Goedicke K, et al (2004) Structure and properties of crystalline titanium oxide layers deposited by reactive pulse magnetron sputtering. Surf Coat Technol 180–181: 538–543.

[54] Wojcieszak D, Kaczmarek D, Domaradzki J, Prociow E, Placido F, et al. (2010) Hardness of nanocrystalline TiO_2_ thin films doped with terbium. In: Students and Young Scientists Workshop, 2010 IEEE International. 86–88. doi:10.1109/STYSW.2010.5714178.

[55] Staiger MP, Pietak AM, Huadmai J, Dias G (2006) Magnesium and its alloys as orthopedic biomaterials: A review. Biomaterials 27: 1728–1734. 16246414 10.1016/j.biomaterials.2005.10.003

[56] Li G, Zhao Y, Pang SS, Li Y (1999) Effective young’s modulus estimation of concrete. Cem Concr Res 29: 1455–1462.

[57] Zhang MH, Gjvorv OE (1991) Mechanical properties of high-strength lightweight concrete. ACI Mater J 88: 240–247.

[58] Gaillard Y, Rico VJ, Jimenez-Pique E, González-Elipe AR (2009) Nanoindentation of TiO_2_ thin films with different microstructures. J Phys D: Appl Phys 42: 145305.

[59] Roy BK, Zhang G, Cho J (2011) Titanium oxide nanoparticles precipitated from low-temperature aqueous solutions: III. thin film properties. J Am Ceram Soc : n/a{n/a.

[60] Socrates G (2004) Infrared and Raman Characteristic Group Frequencies: Tables and Charts. Chichester, England: Wiley, 3rd edition.

[61] Wang A, Edwards JG, Davies JA (1994) Photooxidation of aqueous ammonia with titania-based heterogeneous catalysts. Solar Energy 52: 459–466.

[62] Habibi Y, Chanzy H, Vignon M (2006) Tempo-mediated surface oxidation of cellulose whiskers. Cellulose 13: 679–687.

[63] Saito T, Nishiyama Y, Putaux JL, Vignon M, Isogai A (2006) Homogeneous suspensions of individualized microfibrils from TEMPO-catalyzed oxidation of native cellulose. Biomacromolecules 7: 1687–1691. 16768384 10.1021/bm060154s

[64] Johnson RK (2010) TEMPO-oxidized nanocelluloses: Surface modification and use as additives in cellulosic nanocomposites. Ph.D. thesis, Virginia Tech. Virginia Tech Digital Library website. Available: http://bit.ly/xnzDOv. Accessed 2012 Mar 1.

