# Supplementary figures and images for: Hard and Transparent Films Formed by Nanocellulose–TiO2 Nanoparticle Hybrids
Source: PLoS One. 2012 Oct 1;7(10):e45828. doi: 10.1371/journal.pone.0045828 (PMC3462202; doi:10.1371/journal.pone.0045828)

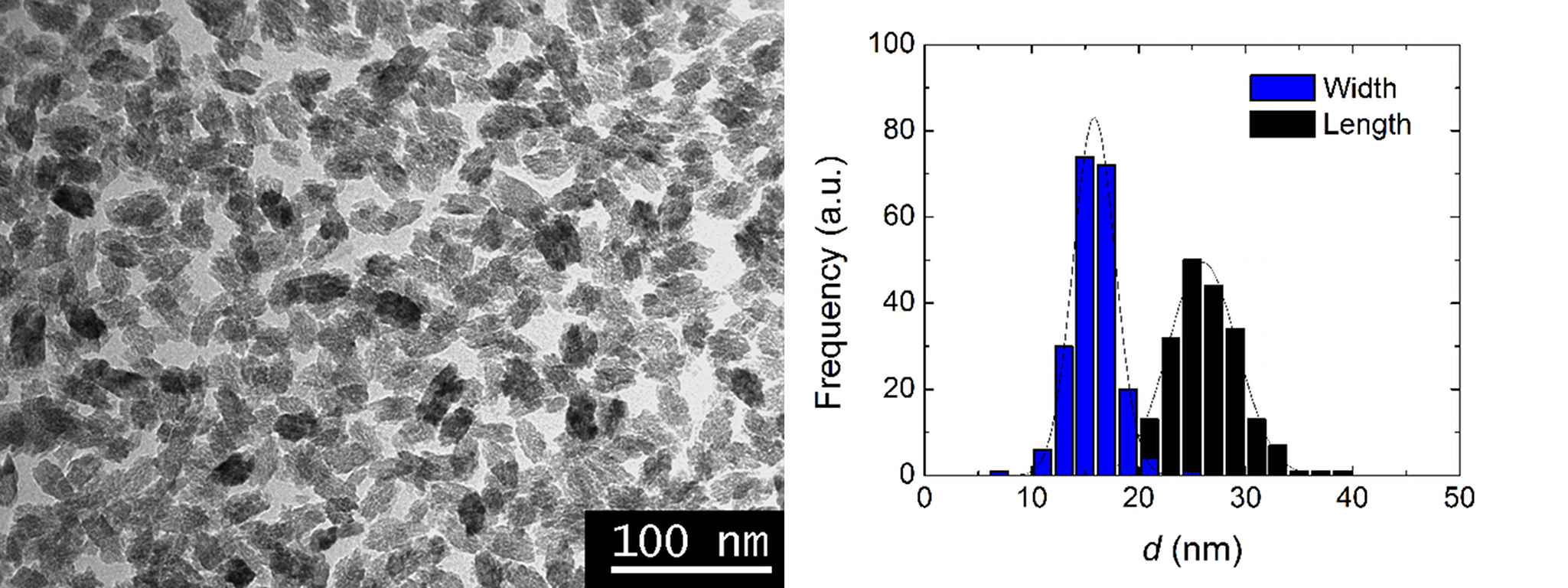

Supplement: Figure S1 — Titanium dioxide nanoparticles and their size distribution. (left) TEM image of titanium dioxide nanoparticles and (right) histograms built from the manual determination of particle length and width. The lines correspond to a fit with a Gaussian distribution function. (TIF) [file pone.0045828.s001.tif]

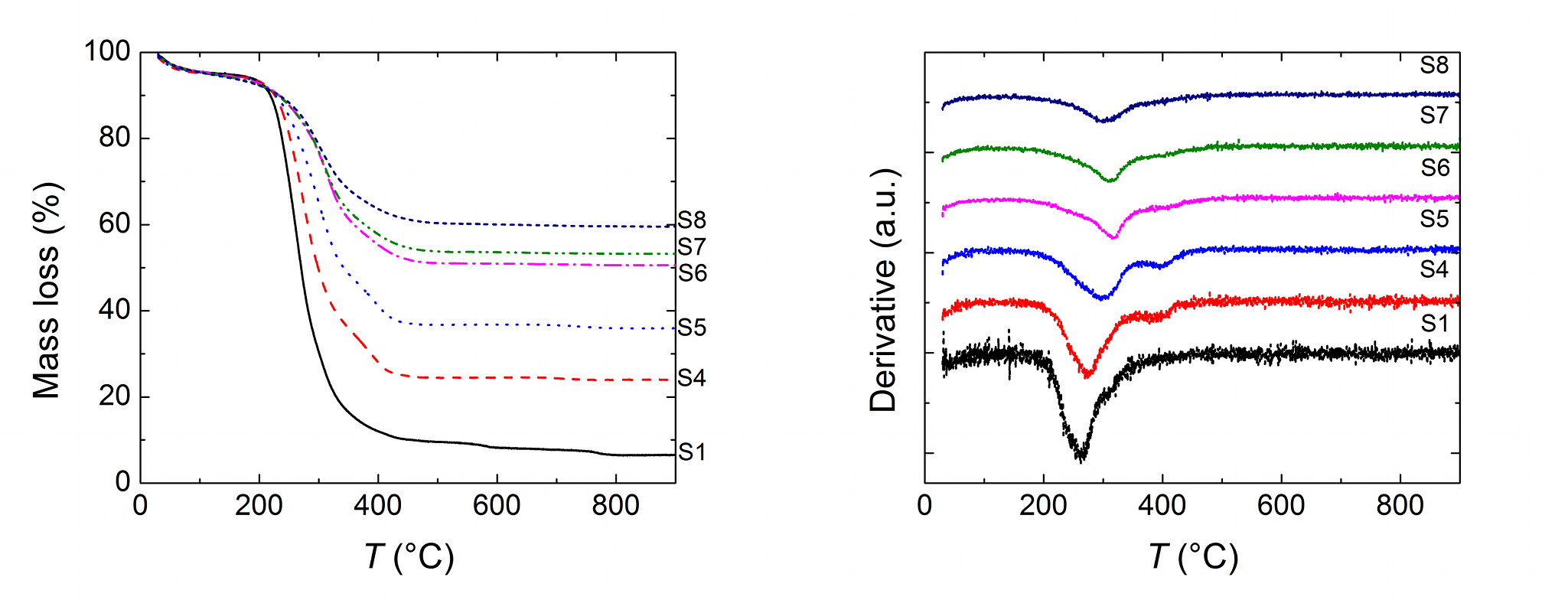

Supplement: Figure S2 — Thermogravimetric analysis of the samples. TGA was performed on a Perkin Elmer Thermogravimetric Analyzer TGA7. Ca. 1 mg of the different hybrids (freeze-dried from the solutions) was filled in a platinum cup and analyzed under technical air from 30–900°C at a heating rate of 5 K/min. (left) The initial weight loss up to 200°C corresponds to the release of adsorbed water. The second weight loss from 200–500°C is due to the removal of NFC. (right) Derivative of the mass loss. (TIF) [file pone.0045828.s002.tif]

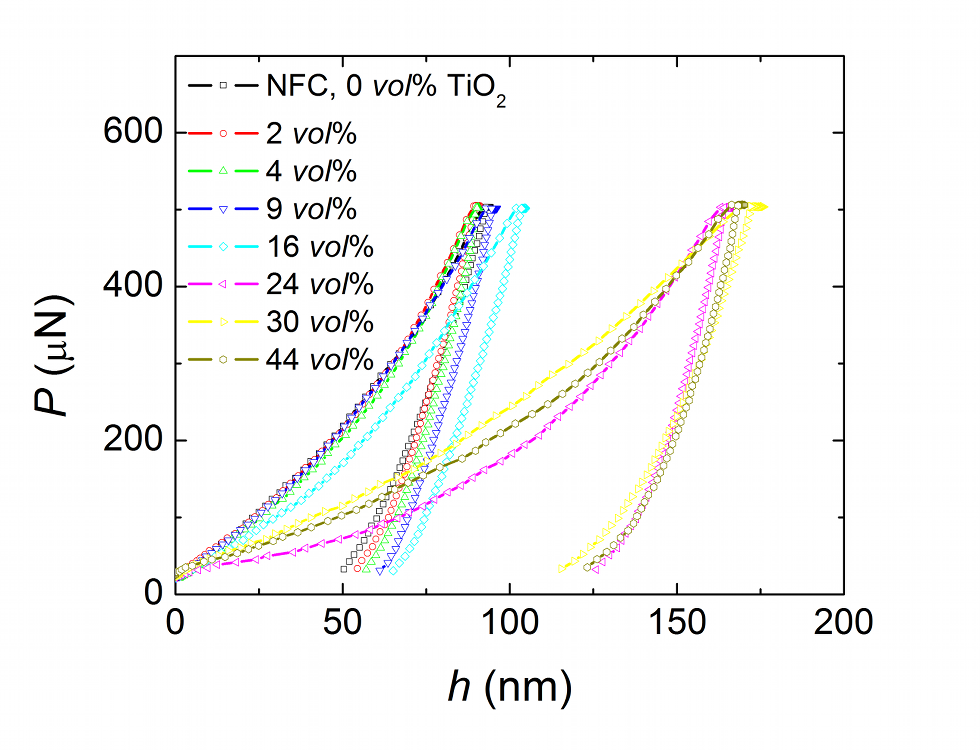

Supplement: Figure S4 — Load – displacement curves. Indentation curves corresponding to hybrids with different compositions deposited as films on glass substrates. The thickness of the films is ca. . (TIF) [file pone.0045828.s004.tif]

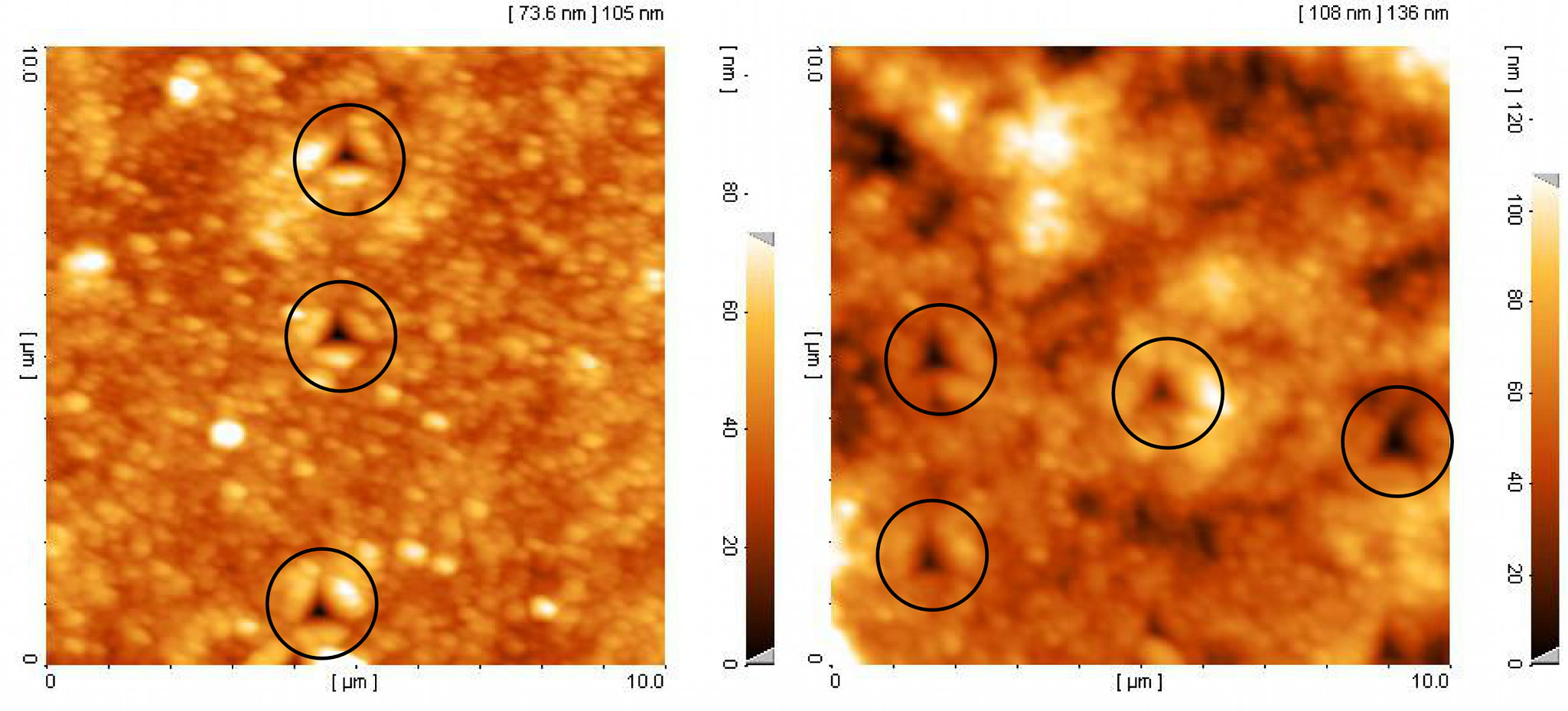

Supplement: Figure S5 — Atomic force microscopy derivative images. Indentations performed on samples S2 (left) and S7 (right). The circles highlight the indentations, where no pronounced pile-up is observed. (TIF) [file pone.0045828.s005.tif]
