# Supplementary material for: Hard and Transparent Films Formed by Nanocellulose–TiO2 Nanoparticle Hybrids
Source: PLoS One. 2012 Oct 1;7(10):e45828. doi: 10.1371/journal.pone.0045828 (PMC3462202; doi:10.1371/journal.pone.0045828)
Supplement: Table S1 — Additional mechanical properties of the hybrid NFC/TiO2 films obtained from nanoindentation experiments. Parentheses indicate the standard deviation in the last digits. (PDF) [file pone.0045828.s006.pdf]

Sample	*vol%* TiO_2	Plastic energy W_p (pJ)	Elastic energy W_{el} (pJ)	Total indentation energy W_{tot} (pJ)	Elastic recovery W_{el}/W_{tot}
S1	0	13.0(2)	10.0(1)	23.0(1)	0.4363(41)
S2	2	11.3(3)	8.3(2)	19.6(4)	0.42347(128)
S3	4	12.5(2)	8.2(2)	20.7(3)	0.39614(110)
S4	9	11.7(3)	9.1(1)	20.8(1)	0.4362(86)
S5	16	13.5(3)	10.3(1)	23.8(1)	0.4327(71)
S6	24	21.8(12)	9.1(5)	30.9(12)	0.2937(208)
S7	30	27.0(8)	10.4(2)	37.4(8)	0.2773(81)
S8	44	26.2(10)	12.2(6)	38.4(19)	0.3173(181)

Table S1. Additional mechanical properties of the hybrid NFC/TiO_2 films obtained from nanoindentation experiments. Parentheses indicate the standard deviation in the last digits.
